# French multicentre clinical evaluation of helical TomoTherapy® for anal cancer in a cohort of 64 consecutive patients

**DOI:** 10.1186/s13014-015-0477-6

**Published:** 2015-08-14

**Authors:** V. Vendrely, B. Henriques de Figueiredo, E. Rio, J. Benech, S. Belhomme, A. Lisbona, E. Frison, A. Doussau, N. Nomikossoff, M. A. Mahé, G. Kantor, J. P. Maire

**Affiliations:** Department of Radiation Oncology, Hospital Haut-Lévêque, CHU Bordeaux, Pessac France; Department of Radiation Oncology, Institut Bergonié, Bordeaux, France; Department of Radiation Oncology, Institut de Cancérologie de l’Ouest, Nantes, France; Department of Medical Physics, Institut de Cancérologie de l’Ouest, Nantes, France; CHU de Bordeaux, Pole de santé publique, Service d’information médicale, F-33000 Bordeaux, France; Department of Radiation Oncology, Hospital La Timone, Marseille, France; Service de Radiothérapie, Hôpital Haut Lévêque, avenue de Magellan, 33604 Pessac Cedex, France

## Abstract

**Purpose/Objectives:**

To assess feasibility and toxicity of Helical TomoTherapy® for treating anal cancer patients.

**Methods:**

From 2007 to 2011, 64 patients were consecutively treated with TomoTherapy® in three centres for locally advanced squamous-cell anal carcinoma (T2 > 4 cm or N positive). Prescribed doses were 45 Gy to the pelvis including inguinal nodes and 59.4 Gy to the primary site and involved nodes with fractions of 1.8 Gy, five days a week. A positional Megavoltage Computed Tomography was performed before each treatment session. All acute and late toxicities were graded according to Common Terminology Criteria for Adverse Events version 3.0. Survival analysis was performed using the Kaplan-Meier method.

**Results:**

Median follow-up was 22.9 months. Fifty-four women and 10 men were treated (median age: 62 years). Nineteen patients (29.7 %) had T2, 16 patients (25.0 %) T3, and 27 patients (42.2 %) T4 tumours. Thirty-nine patients (60.9 %) had nodal involvement. Median tumour size was 45 mm (range, 10–110 mm). Seven patients had a colostomy before treatment initiation. Fifty-seven patients received concomitant chemotherapy (5-FU/cisplatin or 5-FU/mitomycin-based therapy). Forty-seven patients (73.4 %) experienced a complete response, 13 a partial response or local recurrence, and 11 had salvage surgery; among these, six became complete responders, three experienced metastatic failure, and two local failure. At least four patients experienced metastatic recurrence (concomitant to a local failure for one patient). The two-year overall survival was 85.6 % (95 %CI [71.1 %–93.0 %]), and the one-year disease-free survival, and colostomy-free survival were 68.7 % (95 %CI [54.4 %–79.4]), and 75.5 % (95 %CI [60.7 %–85.3 %]) respectively. Overall survival, disease-free survival and colostomy free-survival were significantly better for women than men (*p* = 0.002, *p* = 0.004, and *p* = 0.002 respectively). Acute grade ≥3 toxicity included dermatologic (46.9 % of patients), gastrointestinal (20.3 %), and hematologic (17.2 %) toxicity. Acute grade 4 hematologic toxicity occurred in one patient. No grade 5 event was observed.

**Conclusions:**

TomoTherapy® for locally advanced anal cancer is feasible. In our three centres of expertise, this technique appeared to produce few acute gastrointestinal toxicities. However, high rates of dermatologic toxicity were observed. The therapeutic efficacy was within the range of expectations and similar to previous studies in accordance with the high rates of locally advanced tumours and nodal involvement.

## Background

Squamous-cell carcinoma of the anus is a rather rare malignancy, accounting for 2 to 4 % of gastrointestinal (GI) malignancies, often occurring in elderly women [1, 2]. However, anal cancer incidence has been increasing over the past decades. Lymphatic spread is frequent (inguinal and iliac nodes) but metastatic evolution is rare [3–5]. Since the publication of Nigro *et al*., the current standard of care consists of chemoradiation, which is highly effective, achieving locoregional control and preservation of anal function without colostomy [6]. Recurrence (20–30 % of patients) is most often locoregional, with more than 80 % occurring within two years of treatment [7–11]. However, delivery of radiotherapy results in significant acute toxicity (dermatologic, GI, hematologic, and genitourinary) because of the large and complex volume to treat and the proximity to critical structures. This important toxicity sometimes leads to treatment interruptions [12]. Therefore, many institutions now use intensity-modulated radiotherapy (IMRT) in order to achieve a better planning target volume (PTV) coverage, and a better protection of organs at risk (OARs), allowing reduction of acute and late toxicities [13–15].

In July 2005, the French National Cancer Institute (INCa) launched a pilot project to study the medical impact of implementing emerging technologies in radiation oncology. This led to the installation in 2007 of three TomoTherapy® (Accuray Incorporated, Sunnyvale, CA) units in France. Before the units were operational, and since no published data on TomoTherapy® was available, treatment indications were validated based on available IMRT literature. Treatment protocols were devised for several diseases including anal canal carcinomas, for which we expected that IMRT using TomoTherapy® could reduce toxicity by effectively dealing with the complex volume to treat [16].

This work is a prospective observational study, reporting the largest published series of patients treated by TomoTherapy® for anal cancer. The primary objective was to evaluate the tolerance to chemoradiotherapy with TomoTherapy®. Secondary objectives were to evaluate overall survival (OS), disease-free survival (DFS), colostomy-free survival (CFS), and prognostic factors of recurrence.

## Methods

We included all consecutive patients treated with TomoTherapy® for squamous-cell carcinoma of the anus between June 2007 and December 2011 in Bordeaux (Bergonié Institute and Bordeaux University Hospital) and in Nantes (René Gauducheau Centre), France. Institutional review board approval was obtained for this pilot study.

### Inclusion criteria

Treatment indications included locally advanced squamous-cell carcinomas, T2 larger than 4 cm, T3, T4 or positive lymph nodes. For these locally advanced cancers, it is recommended to treat inguinal and iliac (internal and external) lymphatic areas. All patients underwent a physical examination including anuscopy with biopsy and rectal examination, a computed tomography of the chest, abdomen, and pelvis; and a magnetic resonance imaging of the pelvis. A positron emission tomography scan was not routinely carried out for patient staging. Only patients presenting a negative serology for Human Immunodeficiency Virus infection were included.

### Chemoradiotherapy

Doses prescribed were 45 Gy to the lymphatic areas at risk (inguinal, mesorectal, and internal and external iliac), and 59.4 Gy to the tumour and the involved nodes with fractions of 1.8 Gy, five days a week. A break in fractions at 45 Gy was introduced in case of toxicity, however this was not mandatory. Chemotherapy involved a combination of cisplatin and 5-FU on weeks one and five. This regimen was the standard regimen for anal cancer treatment in France at that time, and was also used for the control arm of another French trial (ACCORD 03 trial) [17].

### Target delineation and TomoTherapy® planning

Patients were in supine position with knee and foot support. CT scans were performed with contiguous 2.5-mm-thick slices with and without contrast infusion. Delineation was performed by a radiation oncologist and included the following target volumes: the gross tumour volume (GTV: primary tumour and involved nodes) and the clinical target volume (CTV: GTV and lymphatic areas at risk: inguinal, mesorectal, and the internal and external iliac areas). Rules for the delineation of the CTV were as follows: delineation of obturator, external and internal iliac lymphatic areas including vessels plus a 7 mm margin modified by exclusion of bones and muscular structures, inclusion of the totality of the mesorectum and presacral area, and inclusion of ischio-rectal fossa and anal margin. Inguinal areas included inguinal vessels and all visible groins. In case of anal margin involvement, perianal skin was included with a wide margin of 2 cm. In case of posterior vaginal involvement, the entire vagina was included in the CTV. A 10 mm isotropic margin was added to CTV and GTV to create PTV1 and PTV2, respectively. The following OARs were delineated: bladder, genitals, femoral heads, and intestines. The dose was prescribed to the median PTV, with 95 % of the volume receiving at least 95 % of the prescribed dose, 98 % of the volume receiving at least 90 % of the prescribed dose, and 3 % of the volume receiving less than 107 % of the prescribed dose. Dose constraints were defined for femoral heads (D2% <40 Gy), bladder (D2 % <45 Gy, V30 < 20 %), genitals (D2% <50 Gy, V40 < 60 %), and intestines (D2 % <50 Gy, V30 < 20 %). The usual planning parameters were a 2.5 cm field width, a 0.3 pitch and a planned modulation factor of 2–2.5. Some additional dummy structures were delineated such as upper and lower volumes to constrain the dose gradient outside the PTV. Dummy volumes were also created between each separate target volumes. During plan optimization, the first priority was PTV coverage, and then the doses to the OARs and normal tissues were lowered as much as possible without compromising PTV coverage. Quality assurance consisted of in-phantom verification and in-vivo dosimetry using plain film and ion chamber measurements before the first fraction of each treatment and before any treatment plan modification to correlate actual dose and planned dose.

### Clinical course and follow-up

All patients were monitored weekly for acute hematologic, dermatologic, GI (diarrhoea and nausea), and urinary toxicity. After completion of the treatment, all patients were evaluated by a radiation oncologist within six to eight weeks, every four months for the following two years, and every six months during the three following years. All acute and late toxicities were graded according to Common Terminology Criteria for Adverse Events, version 3.0 (CTCAE V3).

### Statistical analysis

Survival was estimated using the Kaplan-Meier method from the date of initiation of radiotherapy. OS was defined as time to death, DFS and CFS were defined as time to progressive disease or recurrence or death and time to colostomy or death, respectively. All patients who did not experience the event of interest were censored at the last follow-up date. Log-rank test was used to determine factors associated with OS, DFS and CFS. Associations between violation of the OAR dose constraints and acute toxicities were estimated using Fisher exact test. All analyses were performed using SAS software (version 9.2; SAS Institute Inc., Cary, NC, USA). A 2-sided *P* value of less than 0.05 was considered statistically significant.

## Results

### Patient and tumour characteristics

**Table 1:** Patient and tumour characteristics (*N* = 64)

Characteristic	Number	Percent
Age, years, median (range)	62	(32–89)
Female gender	54	(84.4)
Perianal involvement	25	(39.1)
Inguinal Node Status		
Negative	45	(60.9)
Positive	19	(39.1)
Vaginal involvement	22	(34.4)
T Category		
T1	2	(3.1)
T2	19	(29.7)
T3	16	(25.0)
T4	27	(42.2)
N Category		
N0	25	(39.1)
N1	15	(23.4)
N2	16	(25.0)
N3	8	(12.5)
M Category		
M0	64	(100.0)
Colostomy prior to treatment	7	(10.9)

Data are shown as number (percentage), except when specified otherwise

Sixty-nine patients were consecutively treated between July 2007 and November 2011 (first day of radiotherapy) for anal cancer: among them five patients presented with metastatic disease and were excluded. In total 64 patients with locally advanced anal cancer were included and analysed. There were 10 men and 54 women; median age was 62 years (range, 32–89 years). Median tumour size was 45 mm (range, 10–110 mm) and 67.2 % of these tumours were T3 or T4 tumours. Sixty-one percent of the patients had lymph node involvement. A colostomy was needed prior to treatment for seven patients (10.9 %) (Table 1).

### Treatment characteristics and dosimetric parameters

**Fig. 1:**
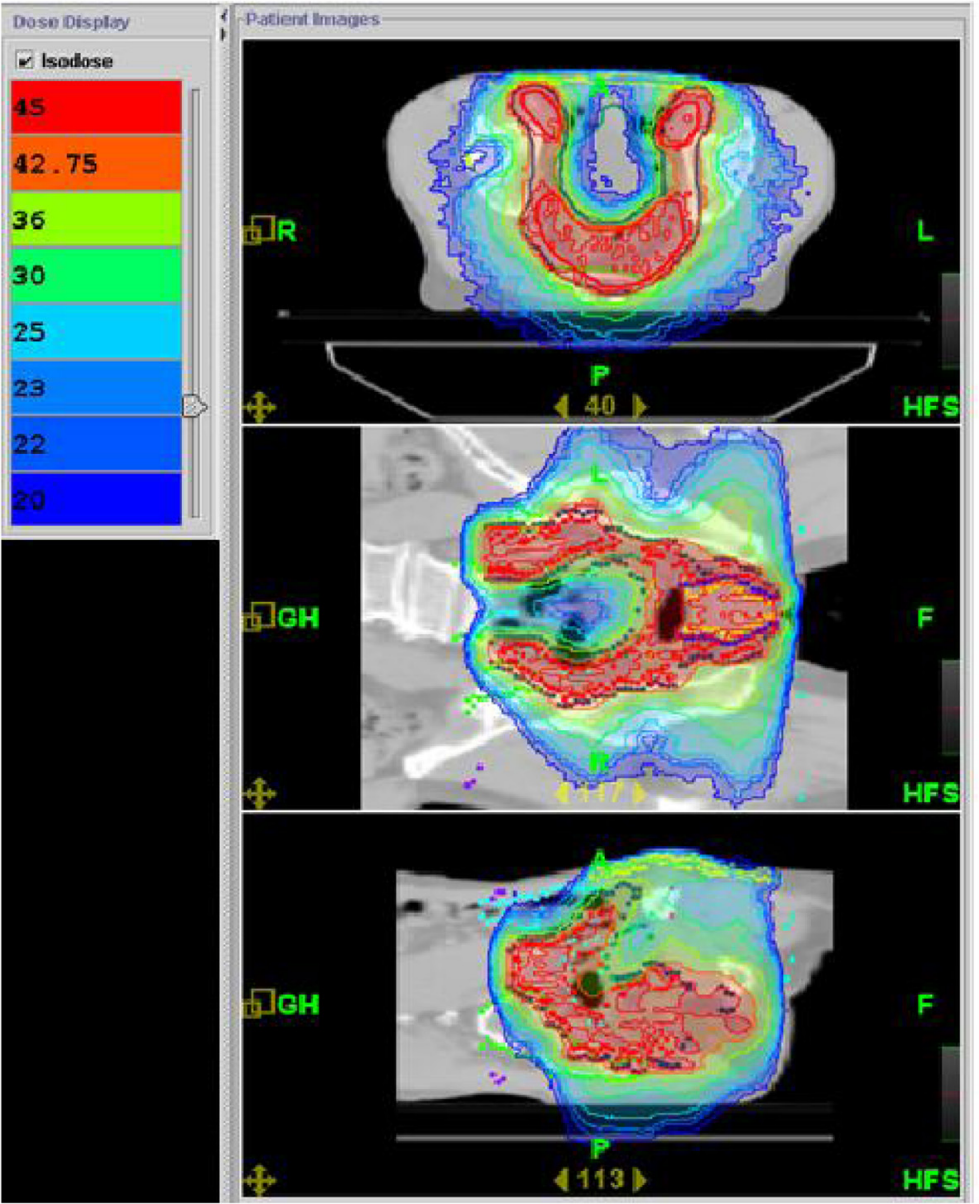
Dose distribution on planning CT with Tomotherapy for the first plan of treatment (45 Gy) for a patient with anal cancer

Median total dose was 59.4 Gy (range, 45.0–67.1 Gy) and median dose per fraction was 1.8 Gy (Fig. 1). Dosimetric parameters concerning PTV coverage and conformity index are summarized in Table 2 and results of dose volume histogram (DVH) analysis for OARs are reported in Table 3. Median overall treatment time (OTT) was 57 days (range, 35–113 days). Thirty patients experienced a treatment break of a mean duration of 5.9 days: in two centres, the treatment break was indicated only based on toxicity for 12 patients whereas in the third centre the break was prospectively planned for 18 patients. Chemotherapy was combined with radiotherapy for 57 patients (89.2 %) and consisted of cisplatin and 5-FU for 49 patients, mitomycine and 5-FU for three patients. For five patients, a combination of eloxatine and 5-FU (folfox regimen) or oral 5-FU in the form of capecitabine were prescribed because of cardiac or renal comorbidities (contra-indication for cisplatin). Seven patients did not receive any concomitant chemotherapy because of comorbidities and age.

**Table 2:** Dosimetric parameters (*N* = 64)

	Mean	(SD)
Planning Target Volume 1		
Dose, Gy	44.04	(5.60)
Conformity Index (opt =1)	1.00	(0.39)
CO (opt =1)	0.93	(0.03)
HI (opt = 0)	0.12	(0.13)
Planning Target Volume 2		
Dose, Gy	60.36	(2.92)
Conformity Index (opt =1)	0.57	(0.39)
CO	0.96	(0.03)
HI	0.07	(0.14)

SD, standard deviation

**Table 3:** Doses to critical organs (*N* = 64)

Dose Volume Histogram for organs at risk	Mean	SD	Minimum	Median	Maximum
Genitalia					
D98 %	15.69	8.39	2.80	13.52	40.23
D2 %	40.53	12.67	18.63	37.94	66.82
Mean D	24.76	9.82	10.48	22.05	58.10
V40 (%)	14.92	23.06	0.05	5.58	98.27
V40 (cm3)	4.78	5.46	0.04	3.55	23.22
Volume (cm3)	78.40	65.62	10.98	58.54	271.2
Right Femoral Head					
D98 %	19.29	7.43	2.42	18.71	47.25
D2 %	39.63	4.86	27.37	40.57	50.07
Mean D	26.90	5.07	15.68	26.47	43.60
Volume (cm3)	112.50	50.98	32.30	122.60	209.20
Left Femoral Head					
D98 %	20.31	6.30	4.12	20.50	40.28
D2 %	39.96	5.02	26.93	40.70	49.36
Mean D	27.38	5.28	15.14	27.44	43.41
Volume (cm3)	108.00	53.02	16.80	114.40	207.40
Bladder					
D98 %	19.78	5.32	10.45	19.40	33.31
D2 %	51.56	5.90	39.20	50.67	64.83
Mean D	32.18	7.39	22.19	30.73	51.63
V30 (%)	50.11	25.12	9.40	46.38	99.80
V30 (cm3)	65.68	61.91	15.33	45.02	357.70
Volume (cm3)	141.9	132.80	45.50	94.51	635.20
Intestine					
D98 %	5.45	5.13	0.50	3.50	19.20
D2 %	45.27	9.17	3.74	45.65	57.47
Mean D	20.64	6.63	1.54	21.69	31.03
V30 (%)	23.50	10.45	4.06	22.17	42.86
V30 (cm3)	219.30	123.00	41.90	187.50	580.60
volume (cm3)	863.20	512.00	231.00	713.50	2295.00

SD, standard deviation

### Clinical outcomes

**Fig. 2:**
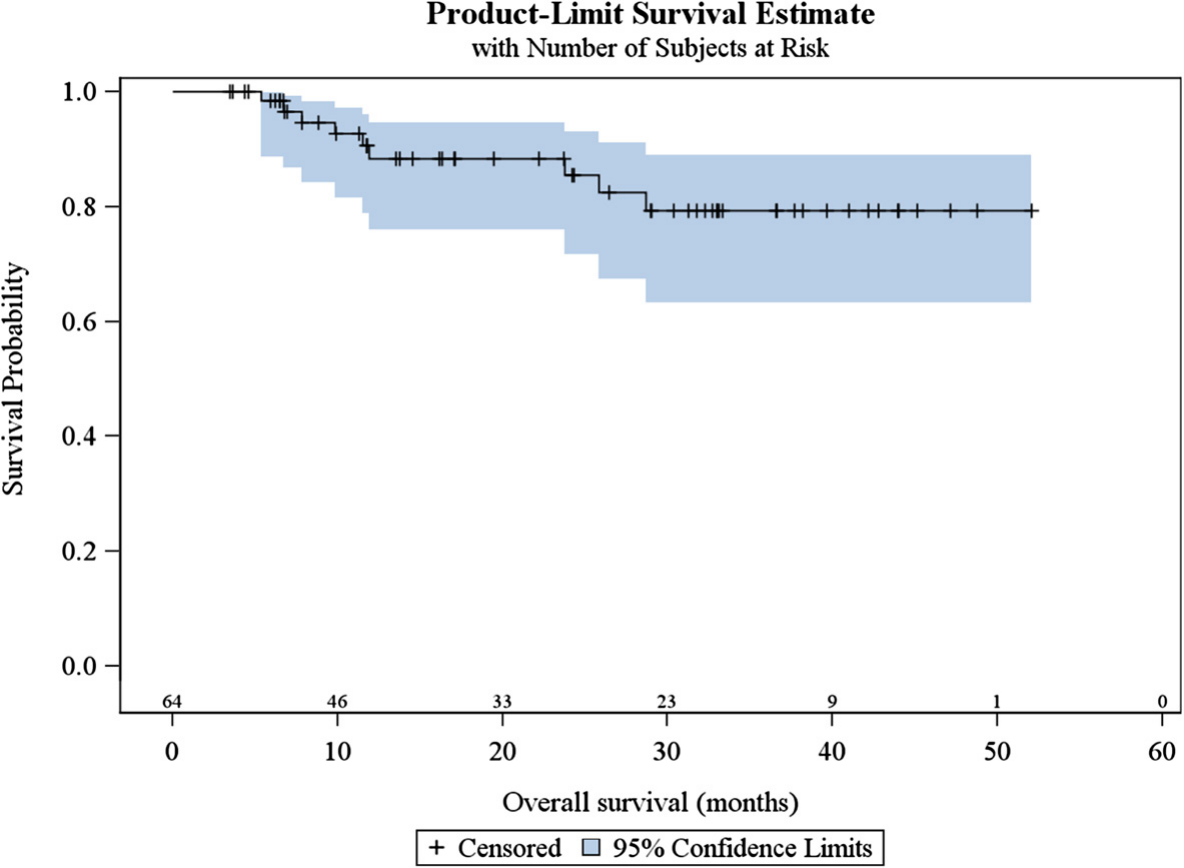
Kaplan Meier estimates of overall survival (*n* = 64)

**Fig. 3:**
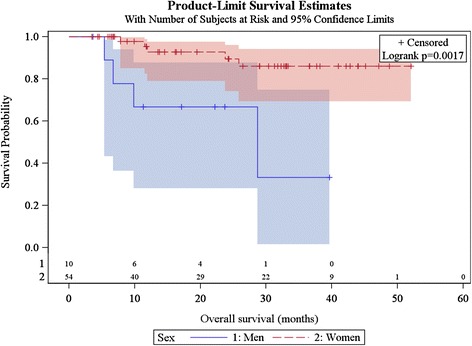
Kaplan Meier estimates of overall survival according to gender (*n* = 64)

Sixty-four patients were evaluated at the first follow-up visit: 34 (54.0 %) had complete response and 30 patients had partial response at the first follow-up visit (median interval, 1.6 months; range, 0.4–5.7 months). Eighteen patients who were considered partial responders at first follow-up evaluation were considered complete responders at the following evaluations (up to 14.2 months after the end of treatment). The median interval between end of radiotherapy and end of follow-up was 22.9 months (range, 3.5–52.0 months). Forty-seven patients (73.4 %) experienced a complete response and 17 patients experienced recurrence after a median 5.4 months following the end of radiotherapy (range, 3.2–9.3 months): three patients had distant recurrence, one patient experienced both local and distant recurrence and 13 patients experienced local failure as first recurrence. Among these 13 patients, 11 were considered partial responders and two were considered complete responders at first evaluation, before diagnosis of disease progression. Eleven patients had to undergo salvage surgery consisting of abdomino-perineal resection: among them, seven were in complete remission, two experienced local failure, one experienced distant failure, and one experienced both local and distant failure. Overall, six patients experienced metastatic failure, and for three patients it was the only recurrence. Among the 17 patients who experienced local or distant recurrence, two patients did not receive concomitant chemotherapy and four patients had a modified chemotherapy regimen (eloxatine based regimen for three patients and capecitabine alone for one patient). Nine patients (14.1 %) died during follow-up, at a median 11.5 months (range, 5.4–28.7 months). One-year OS was 88.4 % (95 %CI [75.9 %–94.7 %]) and two-year OS was 85.6 % (95 %CI [71.1 %–93.0 %]) (Fig. 2). OS was significantly better for women than men (*p* = 0.0017) (Fig. 3). OS was not associated with age, tumour size, nodal status, break in treatment, concomitant chemotherapy or overall treatment time (longer or shorter than 56 days).

**Fig. 4:**
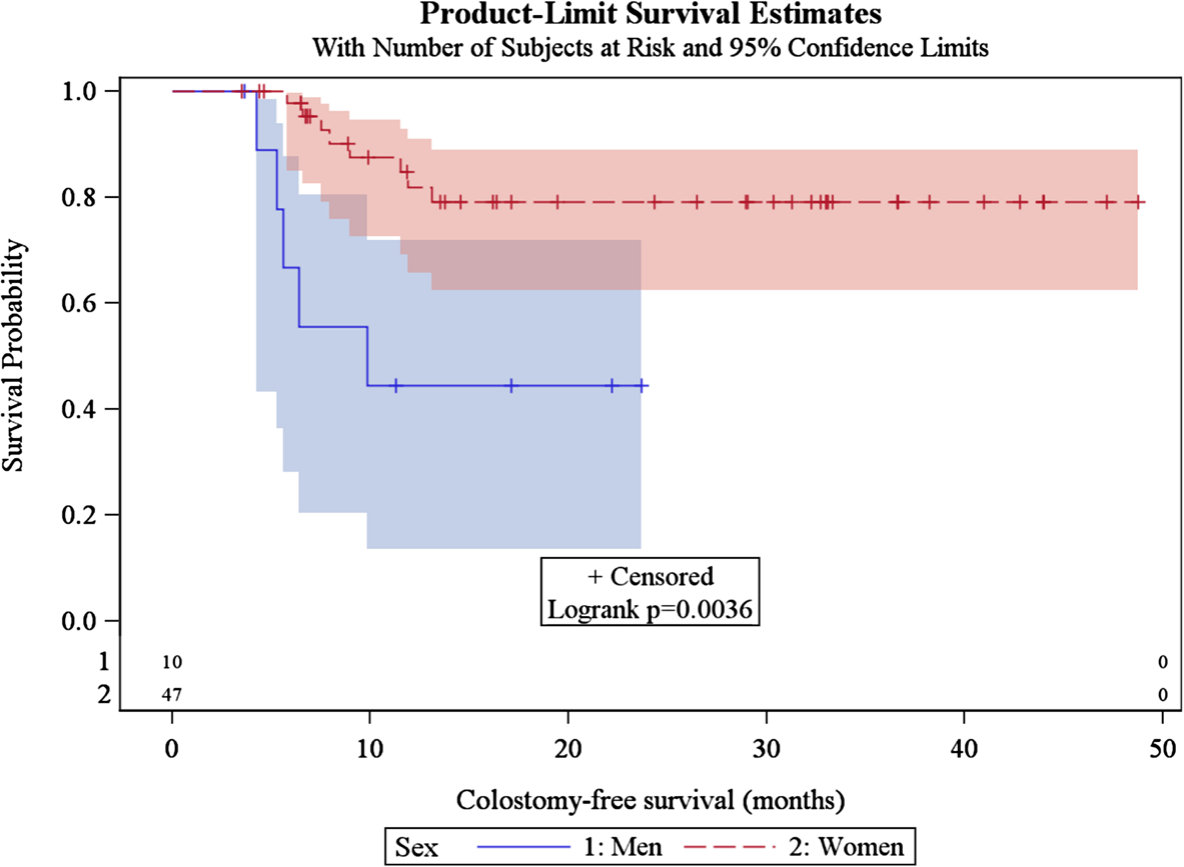
Kaplan Meier estimates of colostomy-free survival according to gender (*n* = 64)

Among the 57 patients without colostomy at inclusion, ten patients (17.5 %) underwent colostomy, at a median of 6.5 months (range, 4.2–13.1 months). One-year CFS was 75.5 % (95 %CI [60.7 %–85.3 %]), and CFS was significantly better for women than for men (*p* = 0.0036) (Fig. 4). There was a trend for increased median age among men (66 years *vs*. 62 for women), there was no difference in terms of size of tumours between genders, there was a trend towards more frequent treatment break among men (60 % of men experienced a break in treatment *vs*. 44 % of women), and 4 men (40 %) did not receive concomitant chemotherapy (*vs*. 5.6 % of women; *p* = 0.01).

**Fig. 5:**
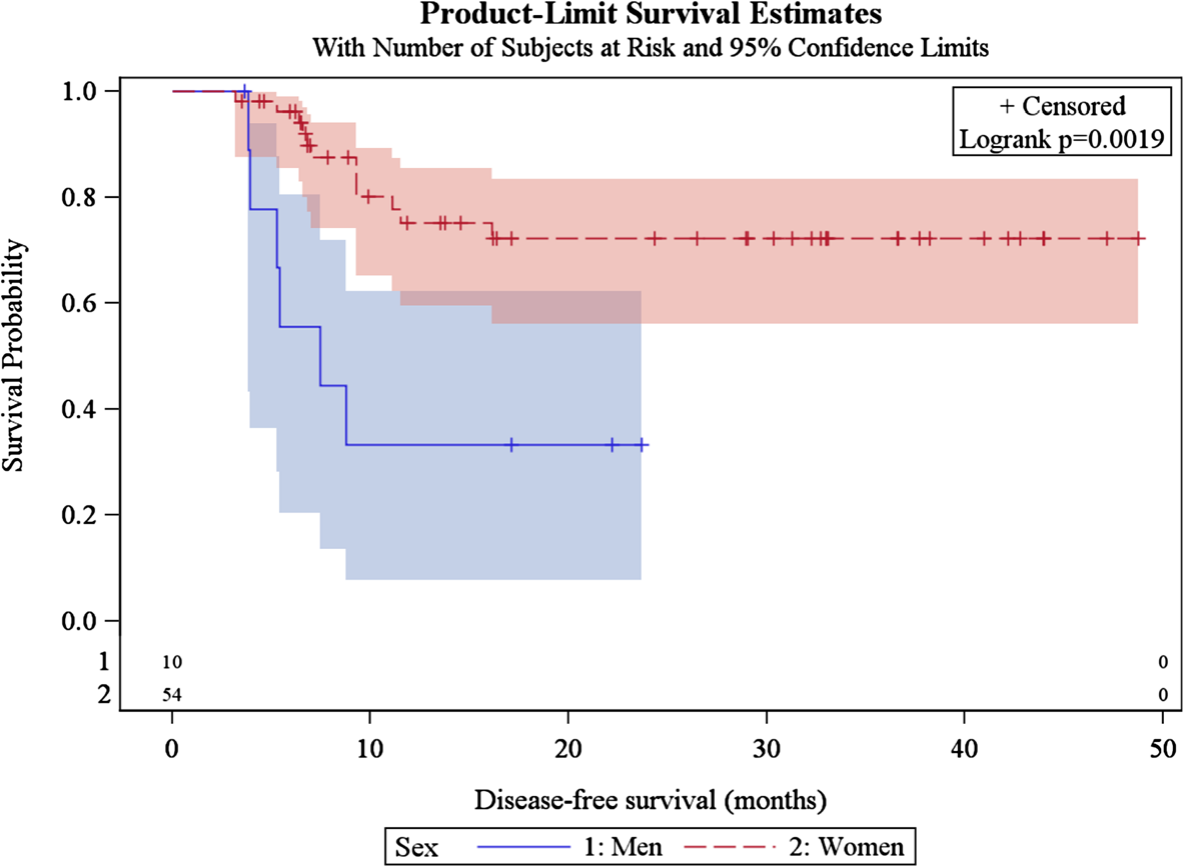
Kaplan Meier estimates of disease-free survival according to gender (*n* = 64)

Seventeen patients (26.6 %) presented progressive disease or recurrence, at a median of 6.8 months (range, 3.2–16.1 months). One-year DFS was 68.7 % (95 %CI [54.4 %–79.4 %]) among the entire population, and women presented a significantly better DFS than men (*p* = 0.0019) (Fig. 5).

### Toxicity

Acute toxicities are summarized in Table 4. A Grade ≥3 acute GI toxicity (such as diarrhoea), occurred in 20.3 %, grade ≥3 of acute skin toxicity (dermatitis) in 46.9 % and grade 3 hematologic toxicity in 17.2 % of patients. One patient experienced an acute grade 4 hematologic toxicity. A break in treatment at 45 Gy became necessary for 30 patients and mean duration of the break was 5.9 days (range, 0–23 days). Analysis of the relationship between violation of the OAR dose constraints and grade 3 or higher acute toxicity indicated a positive association between bladder V30 > 20 % and occurrence of an acute grade 3 bladder toxicity (83.3 % *vs*. 0.0 %; *p* = 0.008).

**Table 4:** Distribution of Acute Toxicity (*n* = 64)

Toxicity Category	Toxicity Grade, n (%)				
	0–1	2	3	4	5
Weight loss	55 (85.9)	8 (12.5)	1 (1.6)	0 (0.0)	0 (0.0)
Nausea / vomiting	54 (84.4)	5 (7.8)	5 (7.8)	0 (0.0)	0 (0.0)
Diarrhea	36 (56.3)	15 (23.4)	13 (20.3)	0 (0.0)	0 (0.0)
Anal mucositis	11 (17.2)	26 (40.6)	27 (42.2)	0 (0.0)	0 (0.0)
Cystitis	56 (87.5)	7 (10.9)	1 (1.6)	0 (0.0)	0 (0.0)
Dermatitis	8 (12.5)	26 (40.6)	29 (45.3)	1 (1.6)	0 (0.0)
Haematological toxicity	47 (73.4)	6 (9.4)	10 (15.6)	1 (1.6)	0 (0.0)

All acute and late toxicities were graded according to Common Terminology Criteria for Adverse Events, version 3.0

## Discussion

Several studies have demonstrated the dosimetric advantages of IMRT over 3D conformal radiotherapy and some studies suggested a decreased toxicity while achieving at least equivalent outcomes [14, 18–20]. The present analysis was performed in the context of the INCa pilot project on the medical impact of implementing new technologies and resulted in a series of 64 patients treated for anal cancer with TomoTherapy® at three institutions. The two-year OS of 85.6 % in our cohort is similar to recent studies, such as Koerber *et al*. (87.4 % in the IMRT group) or Vieillot *et al*. (89 %) [15, 21]. However one-year DFS of 68.7 % could seem low compared to recent results in the literature: it is important to note that our study included locally advanced anal cancers and that two thirds of tumours were T3–T4 tumours, and 61 % were node positive. One could consider that these results are in the expected range given the high rate of locally advanced tumours and nodal involvement. However the question remains about role and type of concomitant chemotherapy since in our study 6 patients among the 11 partial responders had no chemotherapy or an adapted eloxatine based regimen because of comorbidities. Furthermore, the Cisplatin based chemotherapy (standard regimen in France at the time of our study) could also be responsible for this poor DFS since superiority of mitomycine and 5-FU has been demonstrated by the RTOG 98–11 trial [8]. The publication in 2012 of the updated RTOG 98–11 trial results has led to change the recommended chemotherapy regimen in France to mitomycine and 5-FU. An interesting point is the significant association of gender to OS, DFS, and CFS. Such a difference has already been highlighted in other trials: male gender has been observed as a poor prognostic factor in RTOG 98–11 trial and more recently by Koerber *et al*. [8, 15]. In our study, 10 patients were men; one was on immunosuppressant treatment, suggesting this patient might have a more aggressive form of anal cancer. Median age was higher for men (66 years *vs*. 62 for women), there was no difference in terms of size of tumours. There were some differences regarding characteristics of treatment that could explain such a survival difference: 4 men (40 %) did not receive concomitant chemotherapy (*vs*. 5.6 % of women; *p* = 0.01) and there was a trend towards more frequent treatment break among men (60 % of men experienced a break in treatment *vs*. 44 % of women).

Concerning toxicity, our results are consistent with results reported by studies involving IMRT [13, 15, 18]. Two grade 4 toxicities were observed: hematologic toxicity for a patient treated with mitomycine-based chemotherapy and dermatitis for another patient. The level of hematologic toxicity we observed was comparable to ACCORD 3 trial and lower than that in RTOG but this toxicity was also in part due to chemotherapy [7, 9, 13, 17]. Severe acute GI toxicity was lower (18 % of patients) compared to the RTOG study (36 % of the patients). We did not encounter a relationship between compliance with the dose constraints and GI toxicity. This could be explained by the heterogeneity of intestinal volumes among patients and the fact that intestinal dose constraints were specified in terms of proportion and not in terms of absolute volume. For instance, in our study, intestinal volumes ranged from 231–2500 cc (mean, 917 cc) depending on the upper level of the CT scan and on whether the colon was also contoured along with the small intestine. The only relationship between following dose constraints and toxicity was found for acute grade 3 bladder toxicity and V30 > 20 %, which is not a usual dose constraint for bladder, but was adopted in order to minimize the dose to the central pelvic zone.

Rates of dermatologic toxicities were high: 46 % of the patients suffered from grade 3 skin toxicity, which seems similar to that reported in other studies except the RTOG 0529 trial [13]. We must take into account the fact that we did not define any dose constraint for the skin, which could be added in the future in order to reduce this toxicity. We could also study the possibility to reduce our set-up margin. However we have to be cautious, because the skin cannot be avoided when nodes are just below it or in the case of anal margin extension, limitation of which could result in failure.

Another crucial point when using TomoTherapy® is the ability to decrease the overall treatment time (OTT) by avoiding treatment breaks. In the study by Franco *et al*., OTT was 44 days (range 37–55) similar to that in the RTOG 0529 trial (43 days; range 32–59), which could have been reduced by the use of IMRT compared to the RTOG 98–11 trial (49 days; range 4–100) [8, 22, 23]. In our study, OTT was 57 days (range 35–113), which is related to the higher doses and delivery in two plans. Thirty patients experienced a treatment break of a mean duration of 5.9 days. In the two centres where the break was decided only in case of toxicity, this break was necessary for 12 patients (26 % of 46 patients) suggesting that TomoTherapy® could allow treatment without break for 74 % of patients. In the study reported by Franco *et al.*, 9 patients underwent a treatment break (17 %) with a shorter mean duration (3.9 days) whereas the treatment break was necessary for 40 % of patients in RTOG 0529 [22, 23]. Several publications have shown a relationship between OTT and survival for squamous cell carcinomas of the uterine cervix, oropharyngeal cancers, and anal cancer [12, 18, 24, 25]. However, we did not observe any relationship between break *vs*. no break or OTT being longer or shorter than 56 days and OS, DFS or CF, similarly to the results reported by Dewas *et al.* [26].

## Conclusions

TomoTherapy® in locally advanced anal cancer is feasible. At our three centres of expertise in anal carcinoma, this technique seems to reduce acute GI toxicity; nevertheless, high rates of dermatologic toxicities remain prevalent. The therapeutic efficacy is in the range of expectation and similar to previous techniques taking into account the high rates of locally advanced tumours and nodal involvement in the cohort.

## Abbreviations

MVCT: Megavoltage Computed Tomography
CTCAE V3: Common Terminology Criteria for Adverse Events version 3.0
CR: Complete response
PR: Partial response
LR: Local recurrence
OS: Overall survival
DFS: Disease-free survival
CFS: Colostomy-free survival
GI: Gastro-intestinal
IMRT: Intensity-modulated radiotherapy
GTV: Gross tumor volume
CTV: Clinical target volume
PTV: Planning target volume
OARs: Organs at risk
INCa: Institut National du Cancer (National Institute of Cancer)
DVH: Dose Volume Histograms
RTOG: Radiation Therapy Oncology Group
OTT: Overall Treatment Time
